# The protein regulator ArgR and the sRNA derived from the 3’-UTR region of its gene, ArgX, both regulate the arginine deiminase pathway in *Lactococcus lactis*

**DOI:** 10.1371/journal.pone.0218508

**Published:** 2019-06-20

**Authors:** Sjoerd Bouwe van der Meulen, Anne Hesseling-Meinders, Anne de Jong, Jan Kok

**Affiliations:** 1 Department of Molecular Genetics, Groningen Biomolecular Sciences and Biotechnology Institute, University of Groningen, Groningen, The Netherlands; 2 Top Institute Food and Nutrition (TIFN), Wageningen, The Netherlands; University of Kansas Medical Center, UNITED STATES

## Abstract

Small regulatory RNAs (sRNAs) and their enormous potential and versatility have provided us with an astounding insight in the complexity of bacterial transcriptomes. sRNAs have been shown to be involved in a variety of cellular processes that range from stress to general metabolism. Here we report that the gene encoding the transcriptional regulator ArgR is immediately followed by the gene of the small regulatory RNA ArgX. The latter is transcribed from its own promoter. The production of ArgX is induced by increasing arginine concentrations and repressed by CcpA. Previously, ArgR was shown to act as a transcriptional repressor of the catabolic arginine deiminase pathway (*arc* operon) by binding in the promoter region of *arcA*. Here we demonstrate that ArgX downregulates *arc* mRNA levels. Furthermore, ArgX putatively blocks the translation of one of the genes in the operon, *arcC1*, a process that would redirect an intermediate in arginine degradation, carbamoyl phosphate, towards pyrimidine synthesis. Our findings exemplify, for the first time, the combinatorial power of a transcription factor and a small regulatory RNA derived from the 3’-UTR region. The regulators ArgR and ArgX share a common target, but act on transcription and on RNA level, respectively.

## Introduction

Post-transcriptional regulation by regulatory RNAs has added huge complexity to gene regulatory networks, which contained until recently mainly information of protein regulators such as transcription factors (TFs). Regulatory RNAs influence gene expression by base pairing to target mRNAs and thereby affect the stability of the transcript or its translation [1]. This process can result in gene activation or -repression [2–4]. Some regulatory RNAs are transcribed from the DNA strand opposite to the coding strand of the gene they regulate and, thus, (partially) overlap with the gene transcript. These so-called antisense RNAs (asRNAs) have the potential to base-pair with the mRNA by means of a perfect match [5,6]. In contrast, small regulatory RNAs (sRNAs) from intergenic regions usually form a duplex with, often, different mRNAs by means of imperfect base pairing. In many bacteria with GC-rich genomes, the RNA chaperone Hfq acts as a mediator to facilitate the binding between sRNA and its target mRNA [7,8]. A study that involved sequencing of Hfq-bound RNAs in *Salmonella typhimurium*, has shown that the 3’-untranslated region (3’-UTR) of mRNAs can harbor functional sRNAs. Such an sRNA can either derive from the transcript via an RNase-mediated processing event or result from transcription from its own promoter. Both the sRNA and the mRNA share a common terminator structure [9]. Only a small number of sRNAs derived from 3’-UTRs have been functionally characterized, such as DapZ from *Salmonella*, which is transcribed from a promoter located in the very end of the open reading frame of *dapB*, a gene involved in lysine biosynthesis. It has been shown that DapZ represses the synthesis of ABC peptide uptake systems such as Opp and Dpp [9]. The *Streptomyces coelicolor* regulatory sRNA s-SodF is derived from processing of the 3’-UTR of the *sodF* gene, encoding an Fe-containing superoxide dismutase (SOD). s-SodF negatively regulates the Ni-containing SOD, SodN, by blocking translation of *sodN* mRNA and also destabilizing the transcript [10]. CpxQ is another example of an sRNA that is formed by transcript processing, in this case of *cpxP* mRNA cleaved by RNase E. The Cpx pathway monitors misfolded inner membrane (IM) proteins. While the protein chaperone CpxP directs misfolded proteins in the IM towards degradation, the sRNA CpxQ downregulates mRNAs of proteins located in the cell envelope [11]. In *Rhodobacter sphaeroides*, the singlet oxygen-induced SorX sRNA is also generated by RNase E cleavage from the 3’-UTR of an mRNA encoding, in this case, an OmpR-type transcriptional regulator. A 116-nt pre-SorX transcript is further processed into the more abundant 75-nt SorX. SorX targets *potA* mRNA which encodes part of a spermidine uptake system. SorX counteracts oxidative stress by down-regulating *potA*, which results in reduced spermidine uptake, thus lowering the sensitivity of the cells to organic hydroperoxides [12]. It was noted that the limited number of characterized sRNAs derived from 3’-UTRs target functions similar to those of their parental mRNAs [13].

In this study, we strengthen this notion by the characterization of an sRNA, ArgX, located in the 3’-UTR of the *Lactococcus lactis* gene *argR*, a regulator in arginine metabolism in this lactic acid bacterium. From a collection of 186 sRNAs we recently identified in the intergenic regions of *L*. *lactis*, ten were located in the 3’-UTRs of mRNAs [14]. These 3’-UTR-derived sRNAs are formed via transcription from their own promoters, as a TEX-treated pool of sequenced RNAs was used in this study, resulting in an enrichment of primary transcripts [15]. It can, however, not be excluded that these sRNAs undergo additional processing.

Arginine is a non-essential amino acid in *L*. *lactis* that can use it as a nitrogen, carbon and energy source. The synthesis in *L*. *lactis* of arginine from glutamate is encoded by the operons *argCJDBF*, *argGH* and *gltS-argE* while arginine catabolism is mediated by a large *arc* operon, *arcABD1C1C2TD2*. The arginine/ornithine antiporter ArcD facilitates arginine uptake. In *L*. *lactis* MG1363 ArcD1 seems to be the main arginine/ornithine exchanger in the arginine deiminase (ADI) pathway, while ArcD2 functions together with ArcT as an arginine/alanine exchanger in another pathway [16]. Arginine is converted via citrulline into carbamoylphosphate, which is further degraded into ammonia and carbon dioxide with production of one molecule of ATP per arginine. Carbamoylphosphate can also be used for the *de novo* synthesis of pyrimidines. The *arc* operon is highly regulated by the transcription factors CcpA, CodY and ArgR/AhrC [17–19]. CcpA represses *arc* and a catabolite responsive element (*cre* site) is present in the promoter region of *arcA*, P_*arcA*_[19]. In addition, P_*arcA*_ contains six ARC boxes, which represent ARG box half sites that are found in the promoters of genes of the arginine biosynthetic pathway. In the absence of or during arginine limitation, the regulator AhrC facilitates the binding of the repressor ArgR to the ARC boxes, which leads to repression of arginine degradation and simultaneous activation of arginine biosynthesis. This mechanism is reversed in the presence of arginine, which acts as a co-repressor and binds to AhrC. ArgR in a complex with arginine-bound AhrC shifts its preference to ARG boxes. In this model, ArgR acts as a DNA binding protein, while AhrC senses and binds arginine [20]. The *argR* gene is located upstream of the *arc* operon, with only the *argS* gene intervening.

Here we show by transcriptome and proteome studies that ArgX affects *arc*/Arc expression, and verified these results using an *arc-sfgfp* fusion in various genetic backgrounds. Furthermore, we examined the promoter of ArgX and show that it behaves strikingly similar to P_*arcA*_; it responds to arginine and is controlled by the transcription factors CcpA and ArgR.

## Materials and methods

### Bacterial growth, plate reader assays and microscopy

Table 1 presents an overview of strains used in this study. *L*. *lactis* was routinely grown as standing cultures at 30°C in CDMPC [21] or M17 broth (Difco, Becton Dickinson, Le Pont de Claix, France) containing 0.5% (w/v) glucose (GM17), and on GM17 agar plates. Chloramphenicol (5 μg ml^−1^) and erythromycin (5 μg ml^−1^) were added when required. Plate reader assays were performed by loading 200 μl of a mixture of CDM medium and cell culture on a 96-wells microtiter plate. Measurements of optical density of the cultures at 600 nm (OD_600_) and GFP fluorescence (excitation wavelength of 485 nm and emission wavelength of 535 nm) were performed in a Tecan F200 (Tecan Group, Männedorf, Switzerland). A Delta Vision Elite microscope (GE Healthcare Europe GmbH, Eindhoven, the Netherlands) and an Olympus MVX10 macroscope (Olympus B.V., Zoeterwoude, the Netherlands) were used for fluorescence microscopy.

**Table 1:** *L*. *lactis* strains and plasmids used in this study.

Strain or plasmid	Relevant phenotype or genotype	Reference
**Strains**		
MG1363	*L*. *lactis* subsp. *cremoris*, plasmid-free derivative of NCDO712	[22]
NZ9000	MG1363, *pepN*::*nisRK*	[23]
MG1363Δ*ccpA*	MG1363, *ccpA* deletion mutant	[19]
MG1363Δ*codY*	MG1363, *codY* deletion mutant	[24]
MGΔ*argR*	MG1363, *argR* deletion mutant	[25]
SVDM2004	NZ9000, ArgX deletion mutant	This work
SVDM2005	SVDM2004, ArgX gene integrated in pSEUDO_10	This work
SVDM2006	MG1363, P_ArgX_-*sfgfp* fusion integrated in pSEUDO_10	This work
SVDM2007	NZ9000, P_ArgX_ with mutated -10 promoter sequence (-10 mut)	This work
SVDM2008	MG1363, P_ArgX_ -10 mut-*sfgfp* fusion integrated in pSEUDO_10	This work
SVDM2009	MG1363, *ΔccpA*, P_ArgX_-*sfgfp* fusion integrated in pSEUDO_10	This work
SVDM2010	MG1363, Δ*ArgR*, P_ArgX_-*sfgfp* fusion integrated in pSEUDO_10	This work
SVDM2011	MG1363, *ΔcodY*, P_ArgX_-*sfgfp* fusion integrated in pSEUDO_10	This work
SVDM2012	Cm^r^, Em^r^, NZ9000 (pSVDM5004; pNZ8048)	This work
SVDM2013	Cm^r^, Em^r^, NZ9000 (pSVDM5004; pSVDM5005)	This work
SVDM2014	Cm^r^, Em^r^, NZ9000 (pSVDM5004; pSVDM5006)	This work
SVDM2015	Cm^r^, Em^r^, NZ9000 (pSVDM5004; pSVDM5007)	This work
**Plasmids**		
pNZ8048	Cm^r^, high copy number cloning vector	[23]
pIL253	Em^r^, medium copy number cloning vector	[26]
pCS1966	Em^r^, *oroP*, integration vector	[27]
pSEUDO	Em^r^, vector for integration in the *pseudo_10* locus	[28]
pSEUDO-GFP	Em^r^, vector for integration of *gfp* fusion constructs in *pseudo_10*	[28]
pVE6007	Cm^r^, plasmid with thermo-sensitive replication	[29]
pJP005	Cm^r^, pNZ8048, with *recT* under control of P_*nisA*_	[30]
pSVDM5003	Cm^r^, pGhost containing P_*nisA*_-*recT*	This work
pSVDM5004	Em^r^, pIL253 containing P_*arcA*_-*arcABD1*-RBS_*arcC1*_-*sfgfp*	This work
pSVDM5005	Cm^r^, pNZ8048 carrying P_*nisA*_-ArgX	This work
pSVDM5006	Cm^r^, pNZ8048 carrying P_*nisA*_-*argR*X	This work
pSVDM5007	Cm^r^, pNZ8048 carrying P_*nisA*_-*argR*(Δstart)X	This work

Cm^r^: Chloramphenicol resistance marker, Em^r^: Erythromycin resistance marker.

### General DNA techniques and *L*. *lactis* strain construction

Plasmid DNA and PCR fragments were purified with the NucleoSpin Plasmid kit and NucleoSpin Gel and PCR Clean-up kit (Machery-Nagel GmbH, Düren, Germany). The enzymes that were used were produced by Fermentas/Thermo Scientific, Vilnius, Lithuania, unless stated otherwise.

The strain with a mutation in the -10 box of the promoter of ArgX was made by applying the recombineering technique [30,31]. To enable recombineering, *recT* was expressed from pSVDM5003. This plasmid was constructed by amplification of the *recT* gene from pJP005 [30], introducing a BamHI and an XhoI site. The BamHI and XhoI digested *recT* gene was then inserted by restricting pVE6007 [29] with the same enzymes, followed by ligation by T4 DNA ligase. For recombineering transformation, 100 μg of phosphorothioate-modified single-stranded DNA oligonucleotide (Biolegio, Nijmegen, The Netherlands) was used, which was introduced via electroporation using a Bio-Rad Gene Pulser (Bio-Rad Laboratories, Richmond, CA) at 2.5 kV, 25 μF and 200 Ohm. Cells with the anticipated mutation were cured from the pVE6007_*recT* (pSVDM5003) plasmid by growing them overnight in non-selective medium (GM17) at 37°C.

*L*. *lactis*ΔArgX was made by double crossover recombination (DCO). To facilitate DCO, the flanking regions of ArgX were amplified; the restriction sites XbaI and PstI were introduced for the upstream region, and PstI and XhoI site for the downstream region. These fragments were then cloned in plasmid pCS1966 [27] by XbaI and XhoI restriction, followed by ligation and used to transform *E*. *coli*. The plasmids were isolated from *E*. *coli* and integrated in the *L*. *lactis* chromosome using erythromycin selection for integration and 5-fluoroorotate counter selection for the excision step, respectively.

The *L*. *lactis* ΔArgX complementation strain was constructed by amplifying a 388-bp fragment containing ArgX and its promoter and inserting it in pSEUDO [28] using the restriction enzymes BamHI and EcoRI. This plasmid was constructed in *E*. *coli*, isolated and integrated into *L*. *lactis* cells using a double cross-over method [28].

The remaining *L*. *lactis* mutants were made using restriction-and-ligation-independent cloning [32], by separately amplifying the vector backbone and the required insert(s) with the polymerase pfuX7 [33], which were subsequently treated with the USER enzyme mix (New England Biolabs, Ipswich, MA). Ligation mixtures were used to transform *L*. *lactis* by electroporation. A transcriptional fusion was made between P_ArgX_ and the gene for superfolder GFP (*sfgfp*) [34] and integrated in the transcriptionally silent *pseudo_10* locus [28] in order to examine the activity of P_ArgX_. Overexpression of ArgX, *argRX* and *argRX* with a mutated start codon (pSVDM5005, pSVDM5006 and pSVDM5007) was done by introducing these genes under the nisin inducible promoter P_*nisA*_ on the high copy plasmid pNZ8048 [23]. To test the effect of the overproduction of these three variations of ArgX on *arc-sfgfp* expression, the region of P_*arcA*_ until the start codon of *arcC1* was fused to the *sfgfp* gene and inserted in pIL253 [26], resulting in pSVDM5004. Table 1 and S1 Table provide an overview of strains and oligonucleotides used in this study, respectively.

### Proteome analysis and mass spectrometry

*L*. *lactis* strains NZ9000 and NZ9000ΔArgX were grown in four biological replicates overnight in GM17, diluted 1:100 in 100 ml fresh GM17 and grown to an OD_600_ of 0.5, after which the cells of 50 ml of culture were harvested by centrifugation for 10 min at 7,200 g and 20°C. Cells were washed twice with 5 ml 20 mM Tris-HCl and re-suspended in 0.5 ml lysis buffer (50 mM Tris-HCl (pH 8.0), 0.3% sodium dodecyl sulfate (SDS), 200 mM dithiothreitol (DTT), 50 mM MgCl_2_, supplemented with DNase I (1 mg/ml), RNase (0.25 mg/ml) and mutanolysin (150 U/ml)), and disrupted with glass beads (75–150 μm, Thermo Fischer Scientific, Rockford, IL) in a Biospec Mini-BeadBeater (Biospec Products, Bartlesville, OK). After a first centrifugation step at 10,000 g for 5 min at 4°C, the resulting supernatant fraction was centrifuged once more (20,000 g 8 min at 4°C). Protein samples were prepared by adding 35 mg/ml urea, 2,5 μl tributylphosphine (Bio-Rad), 5 μl ampholytes (Bio-lyte, Bio-Rad) and 5 mg CHAPS (3-[(-cholamodopropyl)-dimethylammonio]-1-propanesulfonate (Sigma-Aldrich, Darmstadt, Germany) to 140 μl of the cell free extract, resulting in a total sample volume of 250 μl.

First dimension electrophoresis (iso-electric focusing) and second dimension sodium dodecyl sulfate polyacrylamide gel electrophoresis (SDS-PAGE) were performed as described before [35]. Differentially expressed protein spots were identified and statistically analyzed using the Delta2D Image analysis program (Decodon GmbH, Greifswald, Germany), excised, de-stained with 50 mM ammonium bicarbonate in 50% acetonitrile/water and dried prior to overnight digestion with 10 μl of 10 ng/μl trypsin. The obtained peptide mixture was purified with ZipTips (Merck Millipore, Darmstadt, Germany) and spotted on a MALDI plate. MALDI-TOF analysis was performed on a Voyager DE Pro (AB Science, Paris, France) and protein identification was done using the Mascot database search on www.matrixscience.com.

### RNA isolation and quality control

RNA was isolated as described before [14]. In short, frozen cell pellets were re-suspended in 400 μl TE-buffer (10 mM Tris, 1 mM EDTA; pH 8.0), and added with 50 μl of 10% SDS, 500 μl phenol/chloroform and 0.5 g glass beads (75–150 μm, Fischer Scientific). The cells were disrupted in a Biospec Mini-BeadBeater using 2 cycles of 45 sec with a 1-min interval on ice. Nucleic acids were recovered by chloroform extraction and treated with DNase I supplemented with RiboLock RNase inhibitor (Fermentas/Thermo Scientific)) for 30 min at 37°C. RNA was retrieved using standard phenol/chloroform extraction and sodium acetate/ethanol precipitation. RNA pellets were dissolved in elution buffer from the High Pure RNA Isolation Kit (Roche Diagnostics, Almere, the Netherlands) and subsequently stored at -80°C. RNA concentration was measured using a Nanodrop ND-1000 (Thermo Fischer Scientific). RNA quality was assessed by checking the integrity of the 16S/23S rRNA and the presence of any DNA contamination on a 1% agarose/1% bleach gel [36].

### Northern hybridization

Separation of total RNA (10 μg) was performed on 8% or 12% polyacrylamide gels in TAE buffer (40 mM Tris, 20 mM acetic acid, and 1 mM EDTA). As a denaturing agent, 1% bleach was used to replace 7 M urea. The separated RNAs were then transferred to a positively charged Zeta-Probe nylon membrane (Bio-Rad), using semi-dry electroblotting (Bio-Rad). RNAs were covalently cross-linked to the nylon membranes at 1200 mJ in a UVC-508 Ultraviolet Crosslinker (Ultra-Lum Inc., Carson, CA). ssDNA oligonucleotides (See S1 Table) were labeled with ^32^P-γATP using Polynucleotide kinase (Fermentas/Thermo Scientific), according to instructions of the manufacturer. The membranes were incubated overnight for hybridization at 42°C in PerfectHyb Plus Hybridization buffer (Sigma-Aldrich Chemie Gmbh, Munich, Germany) with 9 μl 0.16 pmol/μl of the labeled probe. Membranes were washed twice in 2x saline sodium citrate (SSC) buffer with 0.1% SDS, after which they were exposed to a Phosphor Screen overnight. A Cyclone Plus Phosphor Imager and OptiQuant software (PerkinElmer, Groningen, NL) was used for imaging.

### RNA deep sequencing and data analysis

RNA samples were sequenced at the Primbio Research Institute (Exton, PA), who performed Ribo-Zero rRNA removal and library preparation using the AmpliSeq kit (ThermoFischer Scientific). The cDNA libraries were sequenced on an Ion Proton sequencer (ThermoFischer Scientific). Raw sequence reads were analyzed for quality, trimmed with a PHRED score >28 and aligned to the genome *L*. *lactis* NZ9000 using Bowtie 2 [37]. RKPM values were used as an input for the T-REx analysis pipeline for statistical analysis to determine differentially expressed genes [38]. For the T-REx analysis, a text file describing the factors, contrasts and classes specifying genes from the *arc* (red) and *arg* (blue) operons were written. These text files, together with the RKPM values are available in S2 Table. The RNA-seq data have been uploaded under GEO accession number GSE104515.

## Results

### ArgX biogenesis and homology in other *L*. *lactis* species

**Fig 1:**
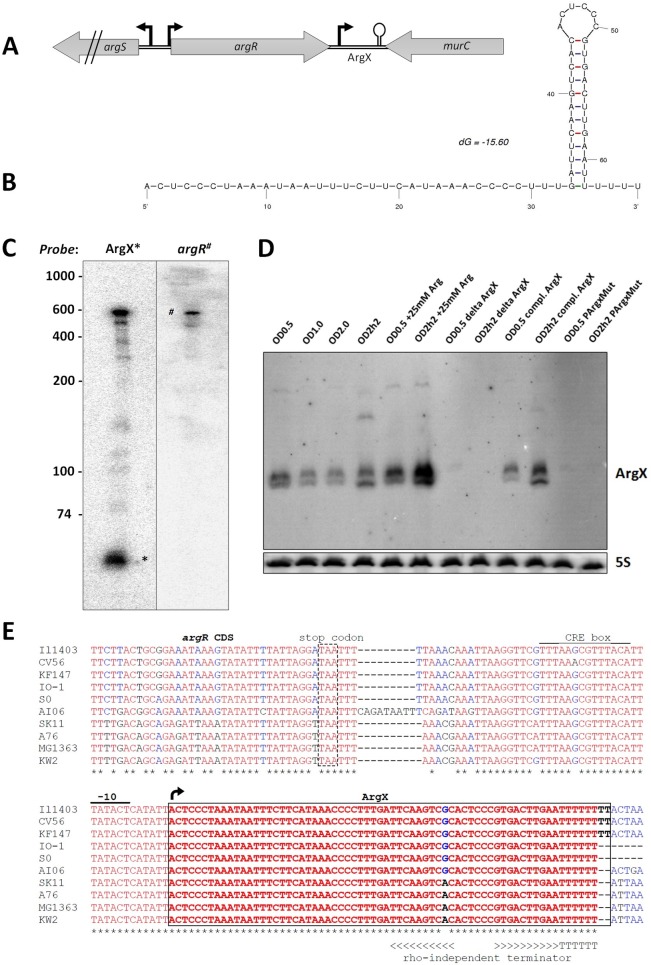
Biogenesis and sequence analysis of ArgX. (**A**) Schematic overview of the genomic locus in *L*. *lactis* of *argR* and ArgX. Black bent arrows indicate promoters. (**B**) Secondary structure of ArgX as predicted by Mfold [40]. (**C**) Northern analysis on an 8% polyacrylamide gel with a probe for ArgX (left) or for ArgR (right). Total RNA was used from exponential (OD_600_ of 1.0) and stationary phase (2h after an OD_600_ of 2.0 was reached) cultures, that were pooled in a 1:1 ratio after RNA isolation. The Northern analysis was repeated twice with identical results. (**D**) Northern hybridization analysis on an 12% polyacrylamide gel of ArgX using various growth phases/conditions and mutants of ArgX, showing that ArgX is derived solely from its own promoter and not from processing. A specific probe for ArgX was used. As a control for RNA quantity and quality, the 5S RNA was used as a control. (**E**) Nucleotide sequence of ArgX (black box) and its promoter region compared to ten *L*. *lactis* species. The black arrow indicates the transcription start of ArgX as determined in *L*. *lactis* MG1363. Asterisks: conserved nucleotides (in red), alternative nucleotides in blue or black, the promoter -10 box is indicated.

Previously, we have identified the putative small regulatory RNA LLMGnc_172 by differential RNA sequencing [14]. This sRNA of ~66-nt, which we rename here as ArgX, is expressed from the same strand as the immediate upstream gene, *argR*, and overlaps with the *argR* 3’-UTR, thus sharing a common terminator sequence (Fig 1A and 1B). Northern analysis using a probe for ArgX shows that an additional larger band exists, which likely represents the *argR* transcript including ArgX. To provide evidence that both ArgX and *argR* overlap, we designed a probe that would anneal to the coding region of *argR*. Since we observed identical sizes on both blots, we conclude that ArgX indeed overlaps with *argR* (Fig 1C).

ArgX was identified in a 5’-enriched fraction of total RNA of *L*. *lactis* that resulted in primary transcript reads due to a treatment with Terminator 5´-Phosphate-Dependent Exonuclease (TEX). A nearly perfect -10 RNA polymerase recognition sequence (TATACT) was present upstream of the ArgX transcription start site [14], indicating that ArgX is transcribed from its own promoter. We mutated the putative -10 promoter sequence to investigate the possibility that ArgX might also be derived from a processing event, as has been described for CpxQ [11]. Northern analysis revealed that ArgX is not produced in the strain with the -10 box mutation and that, thus, ArgX is derived only by activity from its own promoter, not by processing from the larger *argX* transcript (Fig 1D). In an *L*. *lactis* ΔArgX complementation mutant, carrying ArgX including a 246-bp region upstream region, a band of the size of ArgX reappeared in the Northern blot (Fig 1D). We also observe an increase of ArgX expression under high arginine conditions, a result that is further examined below.

A GLASSgo search [39] revealed that ArgX is highly conserved in other strains of *L*. *lactis*, especially when comparing the *argR* coding region immediately upstream of the ArgX gene (Fig 1E). Notably, *L*. *lactis* subsp. *lactis* strains differ from *L*. *lactis* subsp. *cremoris* strains by an A to G mutation in the stem of the terminator (Fig 1E), although this change does not alter its structure or stability, as predicted by Mfold [40]. We could not identify homologs in other, more distant bacteria, using BLAST search and the GLASSgo output.

### The promoter of ArgX is highly regulated and responds to arginine

**Fig 2:**
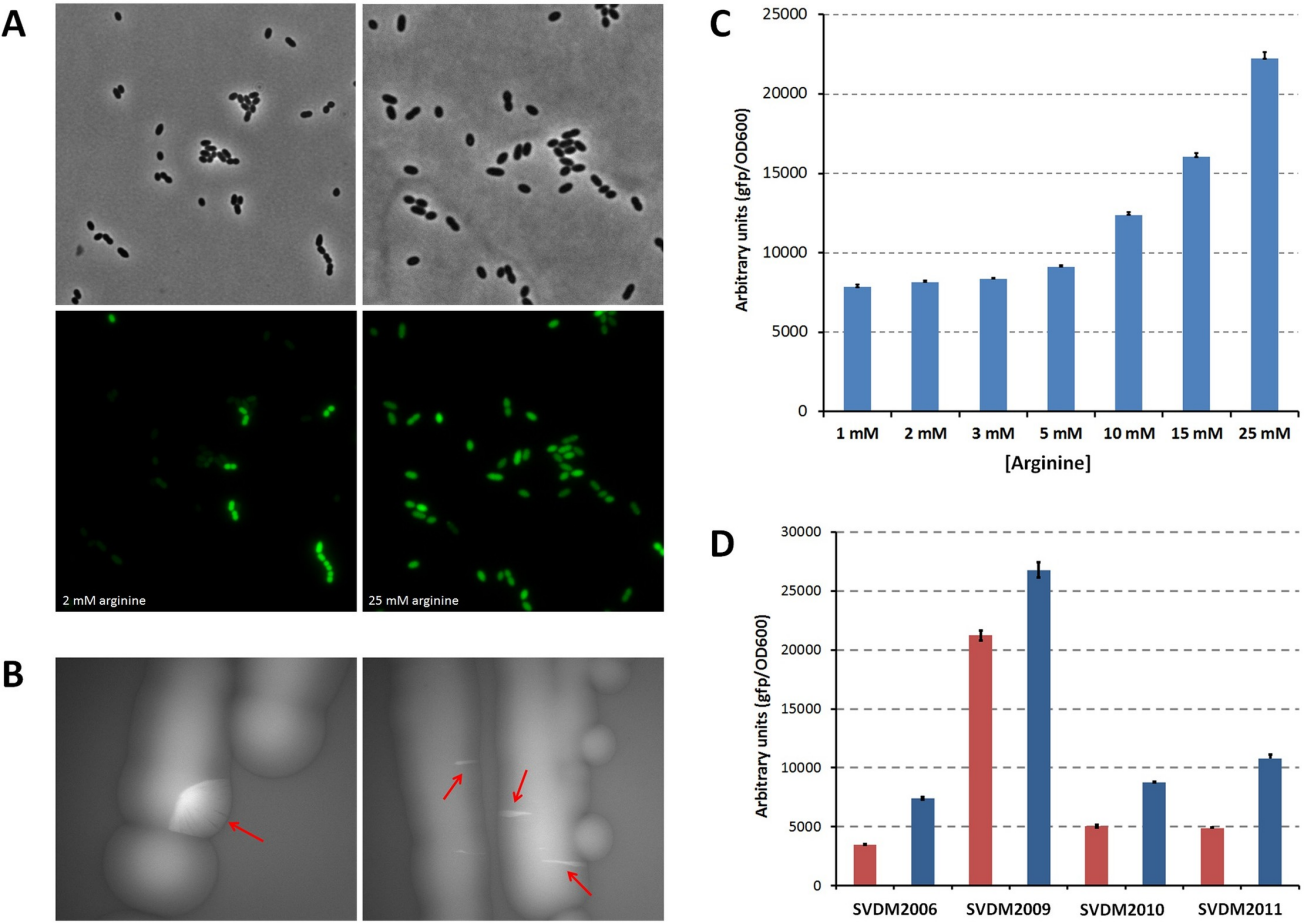
Analysis of ArgX promoter. (**A**) Phase contrast (top) and fluorescence microscopy images (bottom) of cells of *L*. *lactis* SVDM2006, carrying a chromosomally integrated P_ArgX_-*sfgfp* fusion, grown under a low (2 mM) or high (25 mM) arginine concentration. The images depicts a representable situation of at least ten random fields of view. (**B**) Macroscopic pictures of colonies of P_ArgX_-*sfgfp* expressing *L*. *lactis* SVDM2006 cells grown on a GM17 agar plate (M17 contains ~1.5 mM arginine). Bright fluorescent patches of cells with a high P_ArgX_ activity are indicated by red arrows. (**C**) Analysis of P_ArgX_-*sfgfp* activity in *L*. *lactis* SVDM2006 cultures growing in CDMPC with the indicated concentration of arginine. The measurements were performed by a plate reader on cells growing in the stationary phase and were executed in quintuples. Standard deviations are indicated in the error bars. (**D**) GFP fluorescence in *L*. *lactis* SVDM2006 (control), SVDM2009 (*ΔccpA*), SVDM2010 (*ΔargR*) or SVDM2011 (*ΔcodY*), all carrying a chromosomal insertion of P_ArgX_-*sfgfp*. The cells were grown to stationary phase in CDMPC with low (1 mM, red bars) or high (25 mM, blue bars) concentrations of arginine. Measurements were performed in triplicates in a plate reader and standard deviations are indicated in the error bars.

Various sRNAs are controlled by transcriptional regulators, as well as the other way around [41]. For instance, the sRNA RyhB, which is involved in iron homeostasis in *E*. *coli*, is repressed by the ferric uptake regulator Fur [42], of which the translation is negatively influenced by RyhB [43]. Other sRNAs are induced by a sigma factor, such as MicA and RybB, which are regulated by RpoE involved in extracytoplasmic stress [44–46]. In our previous work we predicted the presence of a Catabolite Repressive Element (CRE-box) in the ArgX promoter, slightly upstream of the -10 sequence [14], suggesting that the carbon catabolite protein, CcpA, controls ArgX expression. CcpA is a pleiotropic transcription factor that acts as a repressor when glucose is available as a carbon source [19,47]. The chromosomal P_ArgX_-*sfgfp* fusion was used to examine the activity of P_ArgX_. A heterogeneous gene expression pattern was apparent in cells growing in the presence of moderate concentrations (2 mM) of arginine. This heterogeneity in P_ArgX_ activity was not observed at high levels (25 mM) of arginine (Fig 2A). This behavior relays to the macroscopic level as very bright patches of GFP-producing cells in otherwise non-fluorescent colonies (Fig 2B). The strain containing P_ArgX_-*sfgfp* was grown in CDMPC with different concentrations of arginine and with glucose as a carbon source. These studies revealed that P_ArgX_ is induced by arginine, in a concentration dependent response (Fig 2C). This effect was only seen in stationary phase, which suggests that CcpA might have a repressive effect during the exponential growth phase.

Subsequently, we performed transcriptome analyses on *L*. *lactis* mutants in which the gene of one of three transcriptions factors, CcpA, CodY or ArgR was deleted from the chromosome. In the latter mutant, ArgX was still intact [25]. The deletion of *ccpA* (+ 3.9-fold) or *codY* (- 3.2-fold) had a significant effect on ArgX expression, whereas ArgR deletion did not (see S3 Table). An arginine-dependent response of P_ArgX_ does suggest involvement of ArgR in regulating ArgX. Using the ARC box sequences (half ARG box sites) of P_*arcA*_ [20] we identified a consensus sequence, WGHATADW, that was used to scan ArgX promoter region. This consensus sequence largely overlaps with the -10 sequence of P_ArgX_. We therefore integrated P_ArgX_-*sfgfp* in the *pseudo_10* locus in the *ccpA*, *argR* or *codY* deletion backgrounds and measured GFP activity in the various strains in the stationary phase in the presence of high (25 mM) or low (1 mM) concentrations of arginine. All mutations have an influence on the expression of P_ArgX_ under both conditions, albeit that removal of *argR* or *codY* has a minimal effect while deletion of *ccpA* has by far the highest impact (Fig 2D). It furthermore appears that the effect of CodY as measured with the transcriptional fusion is not in line with the decreased expression of ArgX in a Δ*codY* mutant that we observed in the transcriptome data. We note here that the difference in media, rich GM17 media for the transcriptome study and CDMPC for the plate reader assay, could be at the basis of the observed difference. Also, it is possible that this discrepancy could be due to potential indirect effects caused by CodY that influence the promoter activity of ArgX.

### Expression of *arc*/Arc is elevated after deletion of the ArgX gene

**Fig 3:**
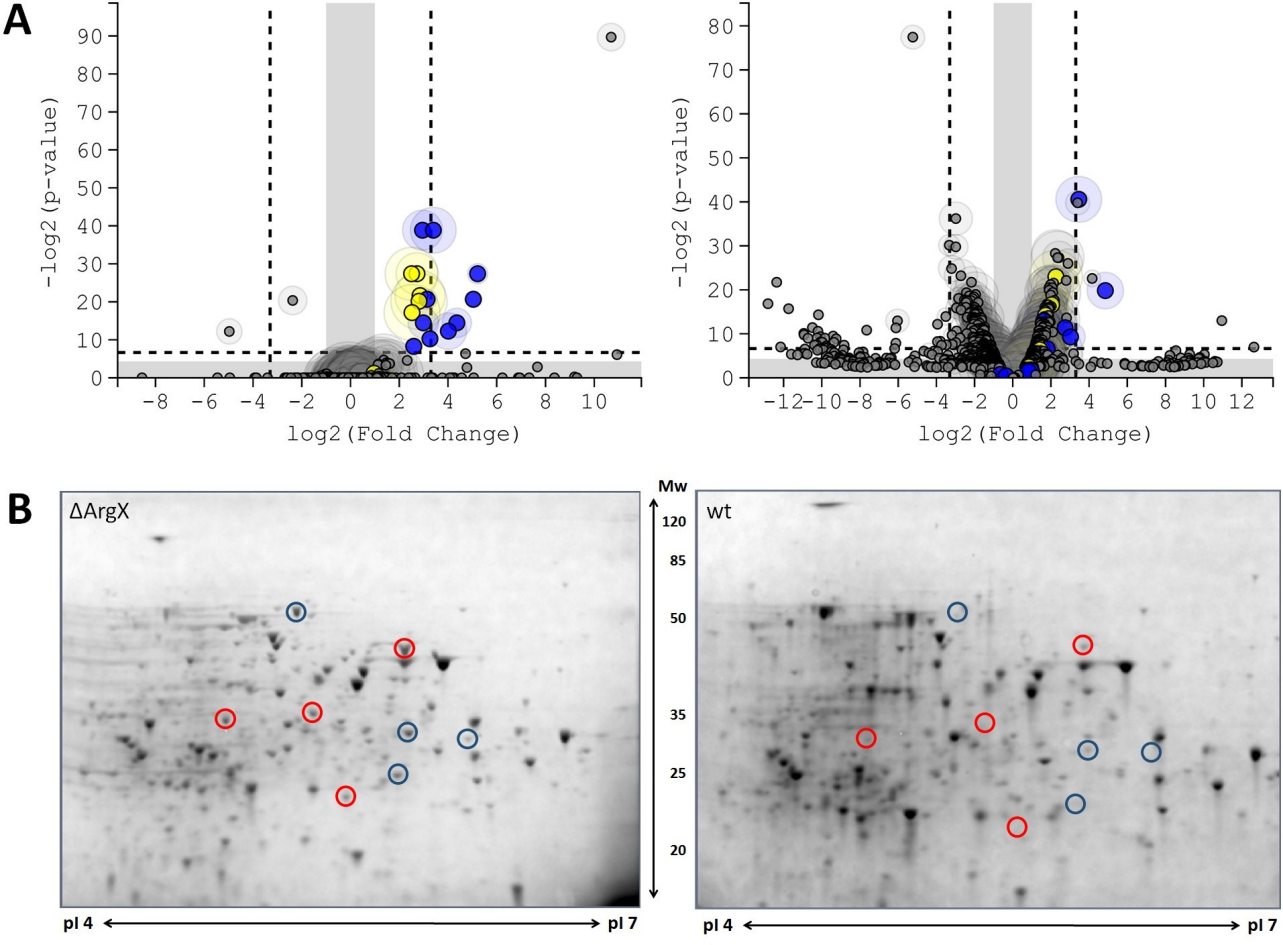
Transcriptome and proteome analysis of the *L*. *lactis* ArgX deletion mutant SVDM2004. (**A**) Volcano plots generated by T-REx [38], showing the RNA-seq results of the effect of ArgX deletion. Genes present outside the grey areas indicate a *p*-value of ≥ 0.05 and a fold change of ≥ 2. Genes outside dashed lines: *p*-value of ≥ 0.01 with fold change ≥ 5. Left: exponential phase, right: stationary phase. Yellow dots represent genes from the arginine catabolism (*arc*), blue dots those involved in arginine anabolism (*arg*). The shaded circles surrounding the genes provides a measure for the expression level. Two biological replicates were used for each strain. (**B**) Analysis by 2D gel electrophoresis of the proteomes of *L*. *lactis* SVDM2004 (left) compared and that of the wildtype strain, NZ9000 (right), grown in GM17 media in four biological replicates. Blue circles represent spots of arginine deaminase (ArcA), red circles represent the ornithine carbamolyltransferase (ArcB) enzyme, as determined by MALDI-TOF analysis.

To examine the potential targets of ArgX, an ArgX mutant strain was constructed that lacks the -10 promoter sequence and the first 32 nucleotides of ArgX (*L*. *lactis*ΔArgX). The mutation was made such that it did not touch the terminator structure in order not to affect transcription termination of the *argR* transcript. The transcriptome and proteome of *L*. *lactis*ΔArgX were compared to those of the wildtype strain *L*. *lactis* NZ9000 using RNA-Seq and 2D gel electrophoresis, respectively. For the analysis, the strains were grown in rich GM17 medium. It is important to mention that no effects were seen at the level of *argR* mRNA or on the ArgR protein level. The *arcABD1C1C2* (6.5 ±0.7), *argGH* (9.3 ±2.1), *gltS*/*argE* (8.5 ±0.6), *argFBDJC* (22.7 ±12.5) and *gltQP* (8.2 ±2.0) gene clusters were upregulated in the exponential growth phase in the mutant strain relative to the wildtype (see S3 Table). The transcripts that encode genes for arginine biosynthesis are possibly induced, since the level of arginine has most probably decreased due to the high expression of *arc* in *L*. *lactis*ΔArgX. One of the most affected genes, with an almost 1500-fold upregulation, was *llmg_1128*. This hypothetical gene has been previously correlated with strain robustness [48], and is possibly expressed by the stress caused by disrupted arginine regulation. Overall, the changes are strikingly similar to those observed in the *L*. *lactis ccpA* and *argR* mutants. In the stationary phase, pleiotropic effects were observed in the strain lacking ArgX, including elevated *arc* expression (3.8 ±0.9) (Fig 3A).

We also studied the proteome to uncover possible differences as a consequence of the absence of ArgX in the exponential growth phase. Eleven protein spots had changed significantly; these were analyzed by MALDI-TOF and identified by using the Mascot server. For three spots, no protein could be identified. Four spots were identified as representing ArcA and another four as ArcB, with an average increase of 4.4 (± 1.5) and 4.9 (± 1.5) fold, respectively, in the ArgX deletion mutant relative to the wildtype strain (Fig 3B). The increase in the amount of ArcA/B proteins reflects the changes of their transcripts but other significant protein changes were not detected.

### ArgX affects *arc* expression directly and decreases growth rate when arginine is absent

**Fig 4:**
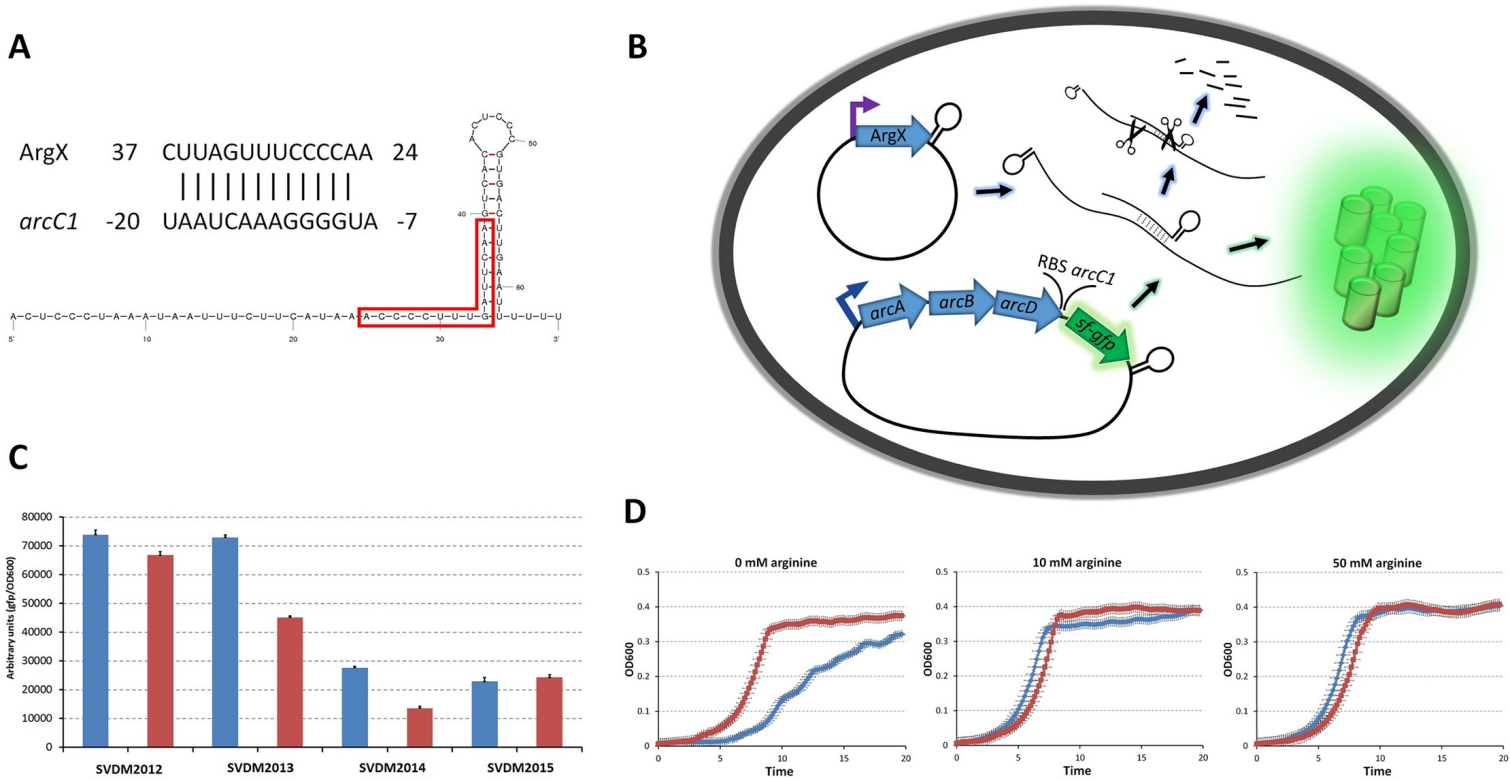
Influence of ArgX/ArgR overexpression on *arc-sfgfp* expression and the effect of ArgX on the growth of *L*. *lactis*. (**A**) RNA duplex between ArgX and the *arcC1* region containing the gene’s RBS, as predicted by TargetRNA2 [49]. The red box in the structure of ArgX shows the region involved in the postulated base pairing between ArgX and *arcC1*. Numbering in *arcC1* counts from the start codon, numbering of ArgX from its TSS. (**B**) Schematic overview of a cell of the *L*. *lactis* strain designed to measure the effect of ArgX/ArgR overexpression on *arc-sfgfp* expression, measured by the development of GFP fluorescence. Lollipops: terminator structure; scissors: RNases; green cages: GFP. (**C**) Results of the experiment described. Blue bars: GFP fluorescence in un-induced cells; Red bars: GFP fluorescence in a culture of cells that were induced with 5 ng/ml of nisin to overexpress ArgX (SVDM2013), ArgR (SVDM2014) and ArgRΔstart (SVDM2015). *L*. *lactis* SVDM2012 is the empty vector control strain. Data derived from cells cultured in CDMPC containing 25 mM arginine, grown in the stationary phase, measured in a plate reader. The experiments were executed in quintuples and standard deviations are indicated in the error bars. (**D**) Growth effect of ArgX deletion mutant in CDMPC medium supplemented by 0.5% glucose and 0, 10 and 50 mM arginine. The red lines represent the deletion mutants of ArgX, blue lines represent the wildtype. Growth curves are the average of five cultures and were executed in a plate reader. The experiment is performed three times with consistent results and the standard error is indicated in the error bars.

ArgX is predicted by TargetRNA 2 [49] to interact with the RBS of *arcC1*, which is the fourth gene encoded on the *arc* transcript, downstream of *arcA*, *arcB* and *arcD1* (Fig 4A). To study the direct effect of overexpression of ArgX RNA on the expression of *arc*, a plasmid was constructed containing a DNA fragment with the *arc* promoter region and *arc* genes until the start codon of *arcC1* fused to the *sfgfp* gene (pIL253::P_*arcA*_-*arcABD1*-RBS_*arcC1*_-*sfgfp*). We decided to construct this fusion on a plasmid because it would otherwise destroy the native *arc* operon. In addition, plasmids were made that contained the ArgX gene or the *argR* gene including the ArgX sequence, the latter with or without a disrupted start codon AAG (*argR(*Δstart*)*X). These genes were each placed under control of the nisin-inducible promoter P_*nisA*_. As a control, the empty expression vector pNZ8048 was used [23] (Fig 4B). The results (Fig 4C) show that induction with nisin of ArgX expression leads to a decrease of *arc* expression. Lower GFP fluorescence was measured under uninduced and induced conditions in strains SVDM2014 and SVDM2015 containing the mutated and the intact *argR* gene, probably because ArgX sRNA is produced from its own promoter in these constructs. Since the media contained 25 mM of arginine, ArgX expression is high. Lowest fluorescence was observed when expression of *argR* was induced. Overexpression of *argR(*Δstart*)*X did not result in a drop of the fluorescent signal in comparison with the un-induced sample.

The expression behavior of *arc* measured by the readout of the GFP in the same plasmid used above, did not show any significant and reproducible differences in the deletion mutant compared to the wildtype strain.

To obtain insight in the biological relevance of ArgX, we grew *L*. *lactis* and mutants of ArgX under different arginine regimes. In the absence of arginine, the ArgX deletion strain grew significantly faster during the exponential phase and reached a higher final OD. By contrast, the same strain grew slightly slower at high arginine concentrations of 10 or 50 mM, while ultimately reaching comparable end ODs (Fig 4D).

## Discussion

### Biogenesis and processing of ArgX

Most trans-encoded regulatory sRNA genes are located in intergenic regions in the chromosome. Recently, a few examples of regulatory RNAs were reported that overlap with the 3’-UTRs of coding transcripts [13]. We have previously identified ten 3’-UTR-overlapping sRNAs in *L*. *lactis* by dRNA-seq. Since the RNA used in this study was enriched for primary transcripts, sRNAs derived from processed 3’-UTRs could not be detected and, if they exist in this organism, remain to be identified [14]. Here we characterized LLMGnc_172 (ArgX), an sRNA that overlaps with the 3’-UTR of the *argR* transcript. ArgX and *argR* use the same terminator sequence. ArgX is expressed from its own promoter as a 66-nt transcript and is not formed via processing of the longer *argR* transcript, as we did not observe any transcripts by Northern analysis after disrupting the -10 sequence in the ArgX promoter. As we observe two distinct ArgX bands on a (12%) polyacrylamide gel it could be that processing takes place at the 5’-end of the ArgX transcript, cleaving off one or only a few nucleotides. Such a processed form of ArgX would carry a monophosphate (5’-P) at its 5’-end while the primary product carries a tri-phosphate (5’-PPP) group. This difference could be biologically relevant as it has been shown in *E*. *coli* that RNase E degrades target mRNAs in response to the 5’-P of the involved sRNA. Moreover, in the absence of its target mRNA, an sRNA that carries a 5’-P is more prone to degradation by RNase E [50]. It is possible that 5’-processing of ArgX would change its molecular function. We did not observe a fixed ratio between the two forms of ArgX by Northern analysis, albeit that the upper band is higher under high arginine availability. Possibly, the ArgX processing itself is regulated and depending on the requirement by the cells for *e*.*g*., arginine or one of its derivatives. Adding to the complexity of the system, both *arc* and ArgX are heterogeneously expressed, at least under “normal” arginine concentrations (1-2mM). This could mean that some cells in a population produce more ammonia by catabolizing the carbamoylphosphate, *e*.*g*. to neutralize the self-produced acid in the environment, while others may invest in the production of proteins and nucleotides using arginine as the precursor. Whether or not such a strategy would provide a benefit for the whole population is interesting to further investigate.

### Regulation of arginine metabolism and the effect of the sRNA ArgX

Arginine metabolism in *L*. *lactis* is regulated by the carbon catabolite repressor CcpA [19], the transcriptional repressor CodY [17] and by the arginine repressor ArgR and its protein partner, AhrC [20]. We show here that ArgX expression is mostly affected by CcpA. The ArgX promoter responds to arginine availability in a strikingly similar fashion as the promoter of the arginine catabolic *arc* operon. This is remarkable as our results show that *arc* is also regulated by ArgX. We hypothesize that the aligned expression could function in arginine homeostasis. An *in silico* prediction using TargetRNA2 hinted at a possible base pairing between ArgX and the RBS of *arcC1*, the fourth gene in the *arc* operon. Deletion of ArgX resulted in an increase in the number of *arc* transcripts as well as a rise in the proteins ArcA and ArcB. Overexpression of ArgX, the *argR* transcript with an intact gene or one in which the *argR* start codon (*argR(*Δstart*)*X) had been mutated all led to a lower expression of *arc*, indicating that ArgX regulates *arc* directly. The possibility that the parental *argR* mRNA is also able to regulate *arc* by a base pairing reaction, was ruled out since we did not observe any decrease in *arc* expression upon overexpression of *argR(*Δstart*)*X in comparison with the un-induced mutant. Overexpression of *argR(*Δstart*)*X leads to a stronger repression of *arc* than when ArgX was overexpressed. This can be explained by the fact that the ArgX promoter is still present in this construct, probably resulting in a very high amount of ArgX transcripts also because of the high level of arginine (25 mM), a condition that was shown to boost ArgX expression. Nevertheless, the repressing effect on *arc* transcription by the protein regulator ArgR appears to be stronger than that of the sRNA ArgX at least under the conditions tested here.

### Arginine, a versatile molecule in carbon and nitrogen metabolism

Arginine has previously been shown to play a role in acid stress tolerance in *L*. *lactis* [51]: The ammonium produced as one of the end products of arginine catabolism can be used to counteract acidification. Ammonium can also be produced for example through conversion of glutamine to glutamate. CcpA represses transcription of the *arc* operon in the presence of abundant glucose during the exponential growth phase [52], while pH neutralization might already be helpful at this stage of growth. Whenever the glucose level drops, and arginine is present, ArgR repression is relieved and *arc* is expressed [20]. The ArgX and *arc* promoters both become activated by increasing amounts of arginine in the stationary phase. The control of *arc* by CcpA suggests that arginine is mainly used as a carbon and/or energy source, at least during the stationary phase. The arginine deiminase pathway imports and catabolizes arginine. However, levels of arginine and its metabolites could rise to undesirable heights, especially if sufficient arginine is available for protein production. The role of ArgX could be to stop the uptake and catabolism of arginine by inducing the degradation of *arc* transcripts. In a less critical situation or perhaps under conditions with high arginine in combination with low glucose, ArgX could redirect the carbamoyl phosphate towards pyrimidine metabolism by blocking translation of *arcC1*, the carbamate kinase that converts carbamoylphosphate into ammonia, ATP and CO_2_. It has been shown that disruption of arginine regulation in *argR* and *ahrC* mutants of *L*. *lactis* increases the activity and gene expression of the *de novo* pyrimidine enzymes PyrE and PyrF [18]. ArgX could function to control the way arginine is utilized by the cell: as a precursor for pyrimidine synthesis or for ATP and ammonia production.

### sRNAs that regulate arginine metabolism in other organisms

Homologs of ArgX were not detected in genomes of other bacterial species. As this search was performed at the level of nucleotide sequence identity we cannot rule out the possibility that ArgX genes are present in other, more distant species. An sRNA was found to be involved in arginine metabolism in *Bacillus subtilis*. In this soil bacterium, the sRNA SR1 blocks the translation of the transcriptional activator *ahrC*, resulting in a decrease of the arginine catabolite gene clusters *rocABC* and *rocDEF* [53,54]. Repression by SR1 on *ahrC* has similar consequences on arginine catabolism in *B*. *subtilis* as does ArgX in *L*. *lactis*, although it takes place at an earlier stage of growth and without the possibility to redirect arginine side-products. Interestingly, SR1, like ArgX, is also repressed by CcpA. Whether the *L*. *lactis* genome harbors an SR1-homolog, whether the *rocR* transcript from *B*. *subtilis* contains an ArgX-homolog or whether ArgX and SR1 are functional homologs are questions still to be answered. In *E*. *coli*, an antisense RNA against the *argR* transcript was identified using dRNA-seq [55]. This transcript, as-*argR* was specifically expressed at an OD_600_ of 0.4, while it was not detected in cells of a culture at an OD_600_ of 2.0. Although no functional analyses were performed, it is expected that as-*argR* targets and helps degrading the *argR* mRNA.

## Conclusions

**Fig 5:**
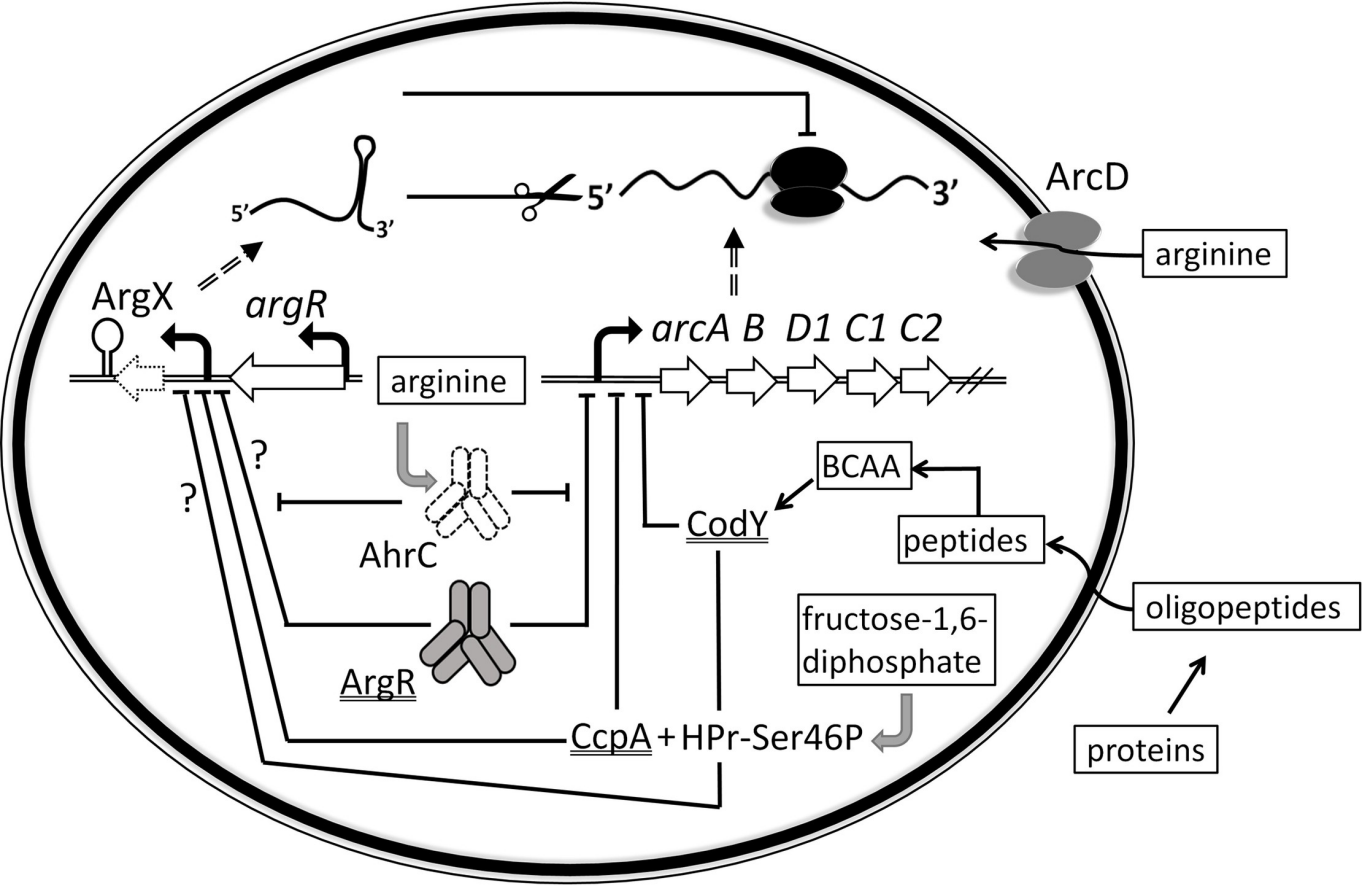
Model of arginine metabolism and its regulation in *L*. *lactis*. Amino acids and (oligo)peptides can be taken up by *L*. *lactis* upon degradation of (milk) protein. CodY senses the intracellular pool of branched chain amino acids (BCAA) and represses *arc* and possibly ArgX expression. CcpA, in combination with Hpr-Ser46P, and ArgR/AhrC repress *arc* and ArgX expression by sensing fructose-1,6-diphosphate and arginine, respectively. ArgX represses *arc* by transcript stability (indicated by a scissor) and/or blocks the translation of *arcC1* (indicated by a black schematic ribosome complex).

The 3’-UTR region of the *L*. *lactis argR* gene, coding the arginine repressor ArgR, harbors a non-coding small RNA regulator gene called ArgX. This sRNA downregulates mRNA levels of *arc*, the operon that specifies the arginine deiminase pathway. ArgX adds another layer to the complex regulation of arginine metabolism. In Fig 5, we present an overview of the current model of arginine metabolism regulation in *L*. *lactis*. Besides regulation of *arc* by various protein transcription factors [17–19], ArgX allows for post-transcriptional regulation by acting on *arc* transcript stability and/or translation of *arcC1*. By blocking *arcC1* translation, carbamoyl phosphate can be directed towards pyrimidine metabolism. We propose that ArgX ensures arginine homeostasis by interfering with the breakdown of intracellular arginine into ammonia, CO_2_ and ATP. The expression of ArgX (and *arc*) depends on relief of carbon catabolite repression by the preferred carbon source via CcpA, and on arginine availability to release repression by ArgR/AhrC. Under low arginine conditions, expression of *arc* is low such that the amino acid can be used for protein synthesis. When arginine is abundant, *arc* is highly expressed. However complete breakdown of arginine might not always be preferred. Re-direction of carbamoyl phosphate towards pyrimidine metabolism allows saving energy. While the conversion of carbamoyl phosphate by ArcC yields 1 molecule of ATP, the production of carbamoyl phosphate for the synthesis of pyrimidines from glutamine consumes 2 ATP molecules. Also, if all arginine would be fully converted, protein synthesis might fall short. We hypothesize that ArgX, of which expression is based on carbon source and arginine availability, can steer arginine towards ATP and ammonia, to pyrimidine metabolism or to protein synthesis.

Altogether, ArgX is a fascinating example of how an RNA from a 3’-UTR region can function as an RNA regulator molecule that is regulated by and intertwined with the function of the gene product of the parental mRNA, in this case *argR*.

## Supporting information

S1 Table Oligonucleotides used in this study. (XLSX)

S2 Table T-REx files for the analysis of *L. lactis* delta ArgX. (XLSX)

S3 Table Transcriptome data obtained for *L. lactis* strains delta ArgX, delta *ccpA*, delta *codY* and delta *argR*. (XLSX)
